# Pathways to Loneliness among Older Adults with Vision Loss: A Machine Learning Study

**DOI:** 10.1093/geroni/igaf122.1741

**Published:** 2025-12-31

**Authors:** Ya-Han Chang, Silvia Sörensen

**Affiliations:** University of Rochester, Rochester, New York, United States; University of Rochester Warner School of Education and Human Development, Rochester, New York, United States

## Abstract

Loneliness is more prevalent among visually impaired than sighted older adults. However, the complex interplay of the factors in shaping loneliness remains unclear. This study applies a machine learning (ML) approach to examine the role multiple variables in predicting loneliness among older adults with age-related macular degeneration (AMD). We used baseline data from adults with AMD (N = 210, Mean Age = 80.1) participating in a depression prevention intervention (Sörensen et al., 2015). We implemented a machine learning decision tree approach to explore the if-then conditions leading to loneliness using emotional responses to vision loss (ERVL), legal blindness, psychological conditions, satisfaction with social life, future planning, optimism, and sociodemographic variables as potential predictors. All variables’ variance inflation factor scores were below 5, indicating a low risk of multicollinearity. Decision tree models with the strongest predictive performance (Train AUC =.88, Test AUC = .78) suggested that legal blindness, ERVL, social life satisfaction, anxiety, depression, and optimism strongly predicted loneliness. Decision trees capture combinations of variables that determine pathways that increase or decrease the risk for loneliness. For example, if social life satisfaction and optimism are low, then the loneliness classification is high (above mean, 24% of cases). If social satisfaction is high and the person is legally blind, then loneliness is low (below mean, 9% of cases). These models effectively capture the relationships between variable combinations that influence loneliness. Decision tree models extend beyond regression to show combinations of variables that describe different if-then pathways leading to loneliness.

